# Supplementary material for: Rapid culture-free diagnosis of clinical pathogens via integrated microfluidic-Raman micro-spectroscopy
Source: Nat Commun. 2025 Dec 16;17:283. doi: 10.1038/s41467-025-66996-y (PMC12783191; doi:10.1038/s41467-025-66996-y)
Supplement: Supplementary file 1 — Supplementary Information [file 41467_2025_66996_MOESM1_ESM.pdf]

Supplementary Information

Rapid culture-free diagnosis of clinical pathogens via integrated microfluidic-Raman micro-spectroscopy

Yuetao Li[1,2,#], Jiabao Xu[1,3,#], Xiaofei Yi[4,5], Xiaobo Li[1], Yanjun Luo[4], Andrew Glidle[1], Phil Summersgill[2], Simon Allen[2], Tim Ryan[2], Xiaochen Liu[6], Wei Yu[7], Xiaobing Chu[7], Shiyu Chen[7], Qian Zhang[7], Xiaogang Xu[5], Xiaoting Hua[6], Qiwen Yang[7], Julien Reboud[1], Yunsong Yu[6*], Wei E. Huang[3*], Jonathan M. Cooper[1*], Huabing Yin[1*]

[1] James Watt School of Engineering, University of Glasgow, G12 8LT, UK

[2] Epigem Ltd, Redcar, TS10 5SQ, UK

[3] Department of Engineering Science, University of Oxford, OX1 3PJ Oxford, U.K

[4] Shanghai D-band Medical Technology Co., LTD, Shanghai 201802, P.R. China

[5] Huashan Hospital, Fudan University, Shanghai 200040, P. R. China

[6] Department of Infectious Diseases, Sir Run Run Shaw Hospital, School of Medicine, Zhejiang University, Hangzhou, China

[7] Department of clinical laboratory, Peking Union Medical College Hospital, Peking Union Medical College, Beijing 100730, P.R. China

[#] Authors contribute equally.

*Corresponding authors: Huabing Yin (huabing.yin@glasgow.ac.uk), Jonathan M. Cooper (Jon.Cooper@glasgow.ac.uk), Wei Huang (wei.huang@eng.ox.ac.uk); Yunsong Yu (yvys119@zju.edu.cn)

Supplementary Figures

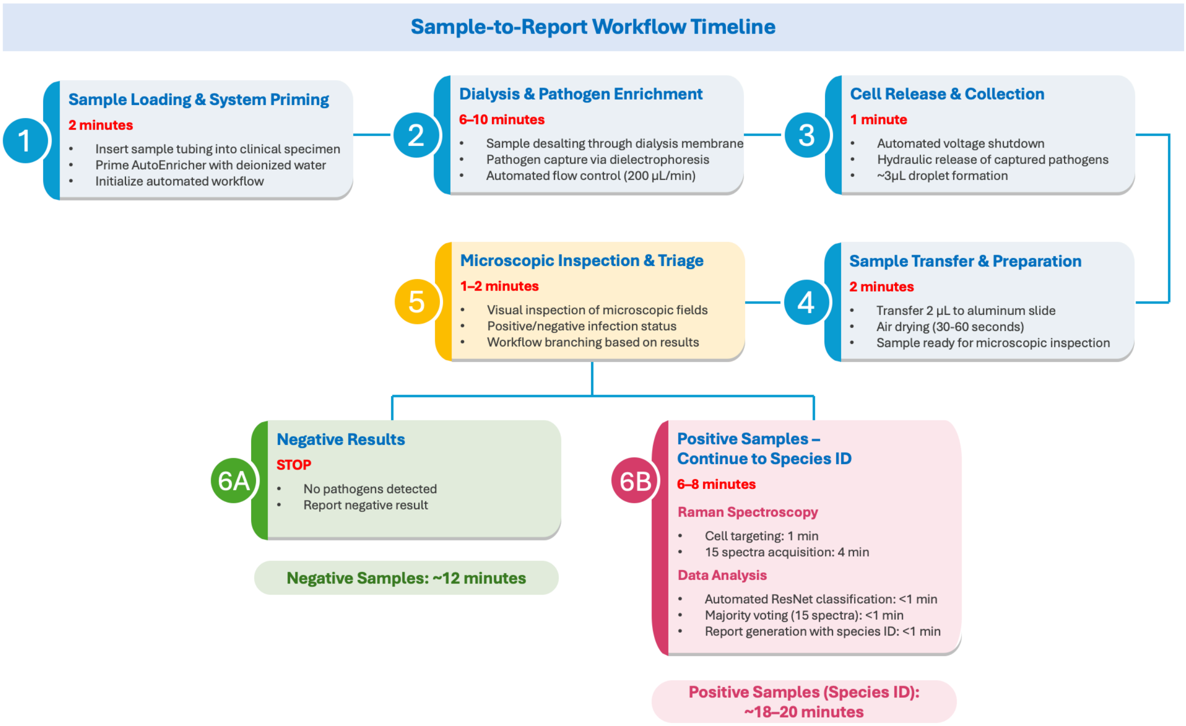

Supplementary Fig. S1 Complete sample-to-report workflow timeline for rapid pathogen identification. The integrated AutoEnricher-Raman spectroscopy platform enables rapid pathogen detection and identification through a branched workflow based on infection status. Steps 1-5 are common to all samples: (1) Sample loading and System Priming (2 minutes): Tubing is inserted into a sample and sterile DI water is delivered into the system at 2 mL/min to remove air. (2) Pathogen Enrichment (~5 minutes): Sample solution is pumped through the disposable dialysis-DEP device where pathogens are captured within the DEP chip. The duration is pre-set based on sample volume and flow rate (e.g., 5 minutes for 1 mL at 200 µL/min). (3) Cell Release (1 minute): Captured pathogens are released through automated voltage shutdown and collected via hydraulic flow into a ~3 µL concentrated droplet. All operations are seamlessly synchronized with customized software (<10 minutes in total for steps 1-3). (4) Cell collection & Transfer (2 minutes): 2 µL of the enriched droplet is pipetted onto an aluminium-covered glass slide and air-dried for 30-60 seconds. (5) Microscopic Inspection & Triage (~2 minutes): Optical inspection determines positive/negative infection status, creating workflow branching. For negative samples (6A): workflow stops (~12 minutes total). For positive samples (6B): Workflow continues with Raman spectroscopy (5 minutes for cell targeting and spectral acquisition of 15 cells) followed by automated ResNet classification (<1 minute) for complete species identification (<20 minutes total).

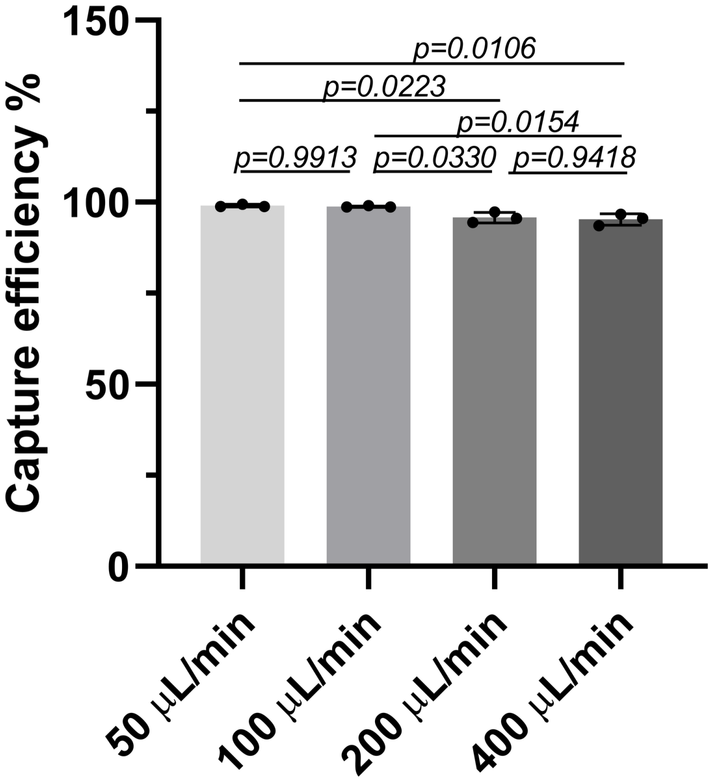

Supplementary Fig. S2 Flow rate optimization for high-concentration bacterial capture. The effect of sample flow rates on capture efficiency using *E. coli* at 3.45×10^7 CFU/mL; AC voltage was fixed at 40V. Data represent mean ± SD from three independent experiments. Statistical significance determined by one-way ANOVA followed by Tukey's multiple comparisons test. Source data are provided as a Source Data file.

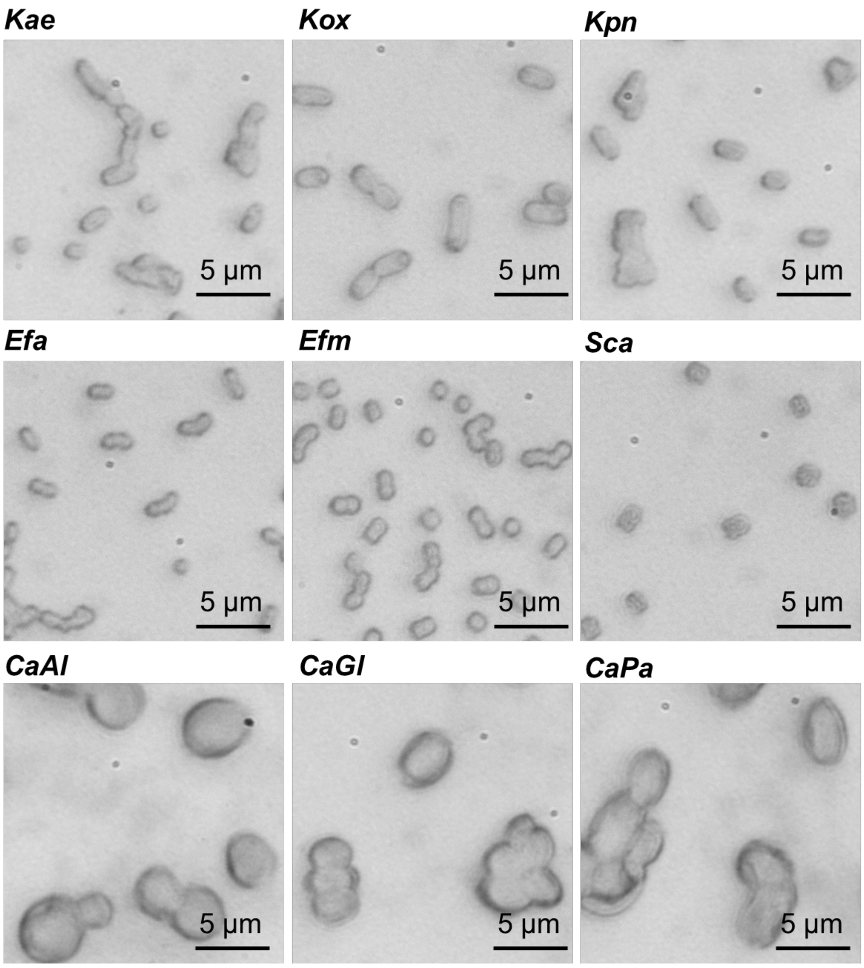

Supplementary Fig. S3 Morphological characterisation of test organisms. Bright-field images of representative bacterial and fungal species used for capture efficiency testing from three independent experiments. *Klebsiella* species display rod-shaped morphology, Gram-positive strains show cocci-shaped morphology, and Candida species exhibit ovoid shapes and mother-daughter budding patterns. Scale bars: 5 µm. Abbreviations: Kae, *Klebsiella aerogenes*; Kox, *Klebsiella oxytoca*; Kpn, *Klebsiella pneumoniae*; Efa, *Enterococcus faecalis*; Efm, Enterococcus faecium; Sca, *Staphylococcus capitis*; CaAl, *Candida albicans*; CaGl, *Candida glabrata*; CaPa, *Candida parapsilosis*.

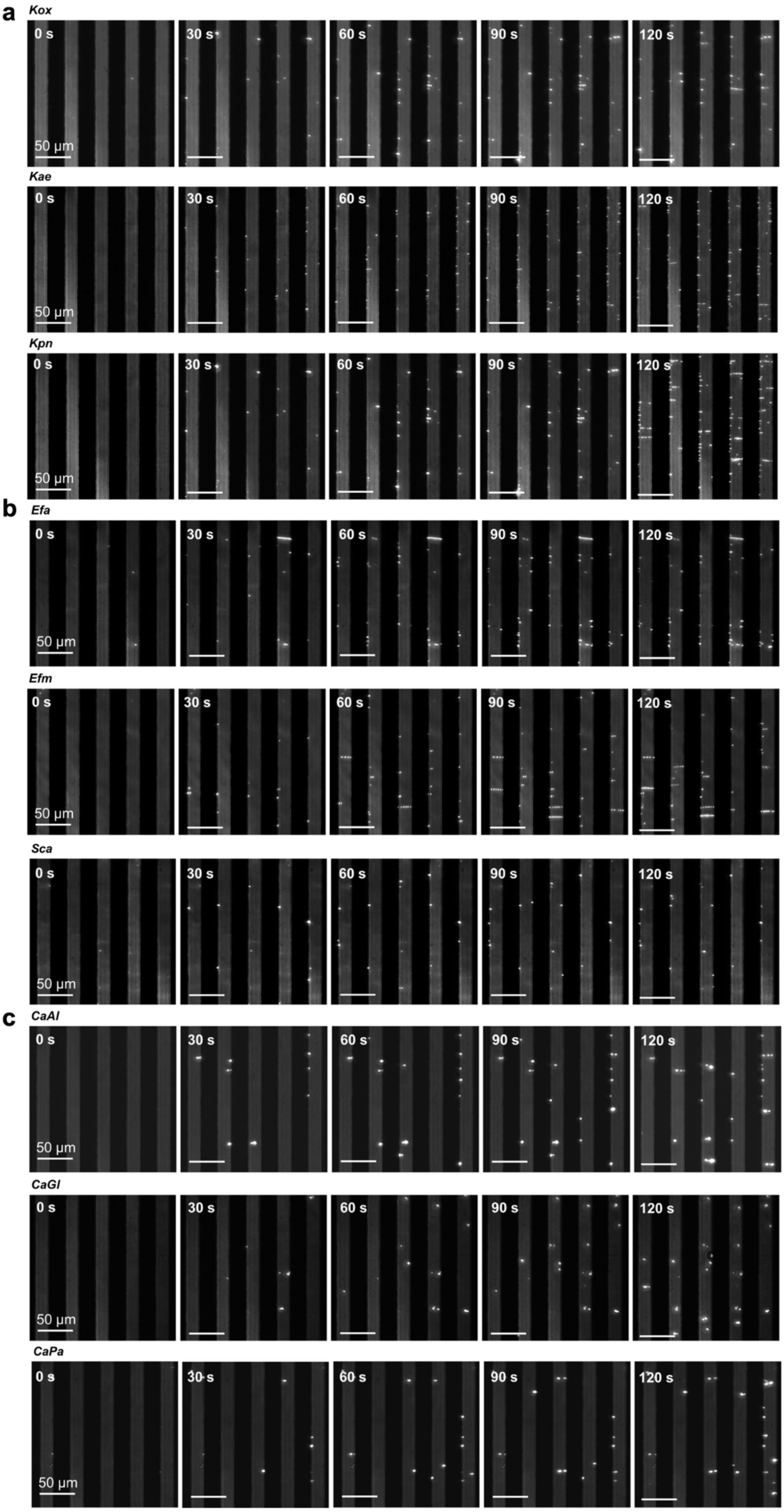
a
Kox
0 s
30 s
60 s
90 s
120 s
50 µm
Kae
0 s
30 s
60 s
90 s
120 s
50 µm
Kpn
0 s
30 s
60 s
90 s
120 s
50 µm
b
Efa
0 s
30 s
60 s
90 s
120 s
50 µm
Efm
0 s
30 s
60 s
90 s
120 s
50 µm
Sca
0 s
30 s
60 s
90 s
120 s
50 µm
c
CaAl
0 s
30 s
60 s
90 s
120 s
50 µm
CaGl
0 s
30 s
60 s
90 s
120 s
50 µm
CaPa
0 s
30 s
60 s
90 s
120 s
50 µm

Supplementary Fig. S4 Time-lapse fluorescence imaging of pathogen capture. Representative sequences from at least three independent experiments showing progressive accumulation of fluorescently labeled **(a)** 3 Gram-negative bacteria, **(b)** 3 Gram-positive bacteria, and **(c)** 3 *Candida* species on DEP electrodes over 120 seconds. AC voltage: 40V, flow rate: 200 µL/min. Scale bars: 50 µm. Abbreviations: Kox, *Klebsiella oxytoca*; Kae, *Klebsiella aerogenes*; Kpn, *Klebsiella pneumoniae*; Efa, *Enterococcus faecalis*; Efm, Enterococcus faecium; Sca, *Staphylococcus capitis*; CaAl, *Candida albicans*; CaGl, *Candida glabrata*; CaPa, *Candida parapsilosis*.

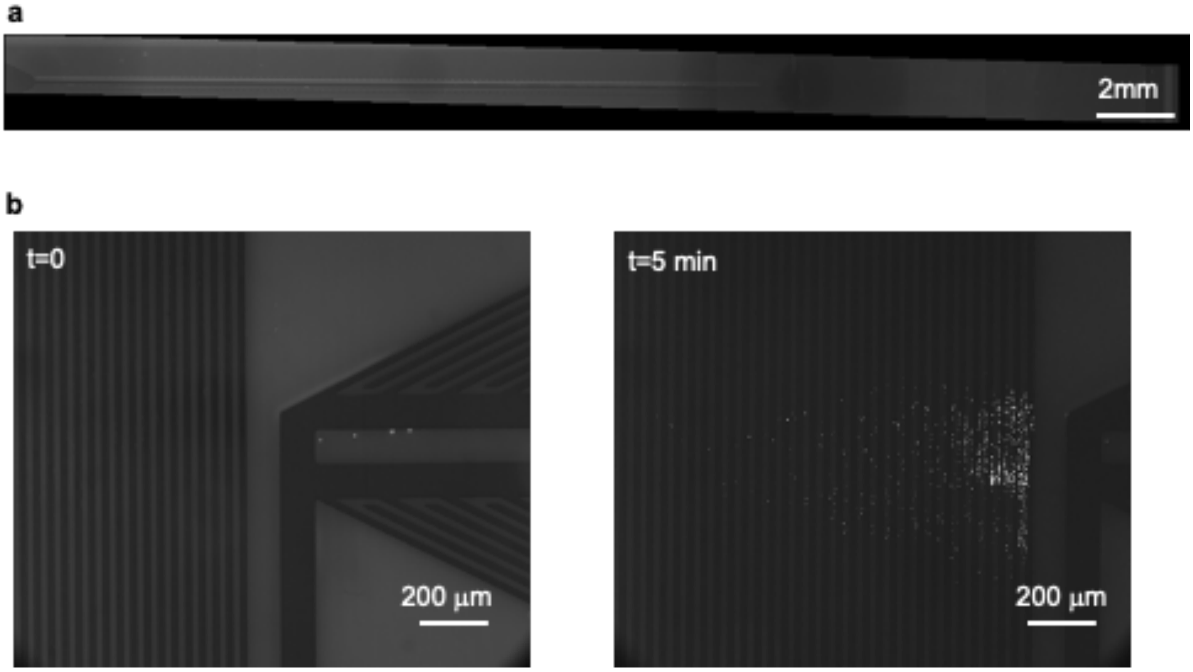

Supplementary Fig. S5 (a) A representative tiled fluorescence image of a DEP device after capturing RFP *E. coli* cells from three independent experiments. The microfluidic channel is 30 mm long and 2 mm wide. The first 20 mm channel consists of a chevron electrode array designed to focus bacteria toward the middle of the channel. The IDT electrode array spans the remaining 10 mm. Most of the RFP *E. coli* cells were captured at the front electrodes. (**b**) A close-up view of the front electrode area before and after trapping REF *E. coli*. Flow rate: 200 µL/min; voltage: 100 kHz, 40 V.

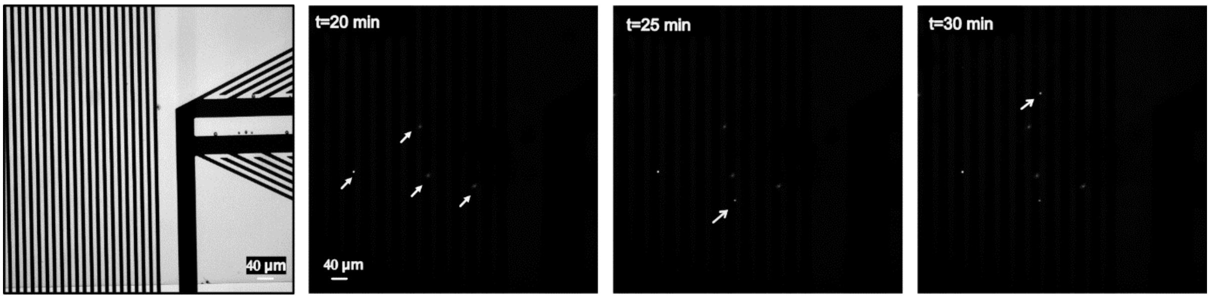

Supplementary Fig. S6 Time-lapse fluorescence images showing the capture of 5 µm PMMA beads at a concentration of 1 bead/ml. Representative from three independent experiments. The sample flow rate was 200 µL/ml, delivering approximately 1 bead every 5 minutes. Voltage: 100 kHz, 40 V.

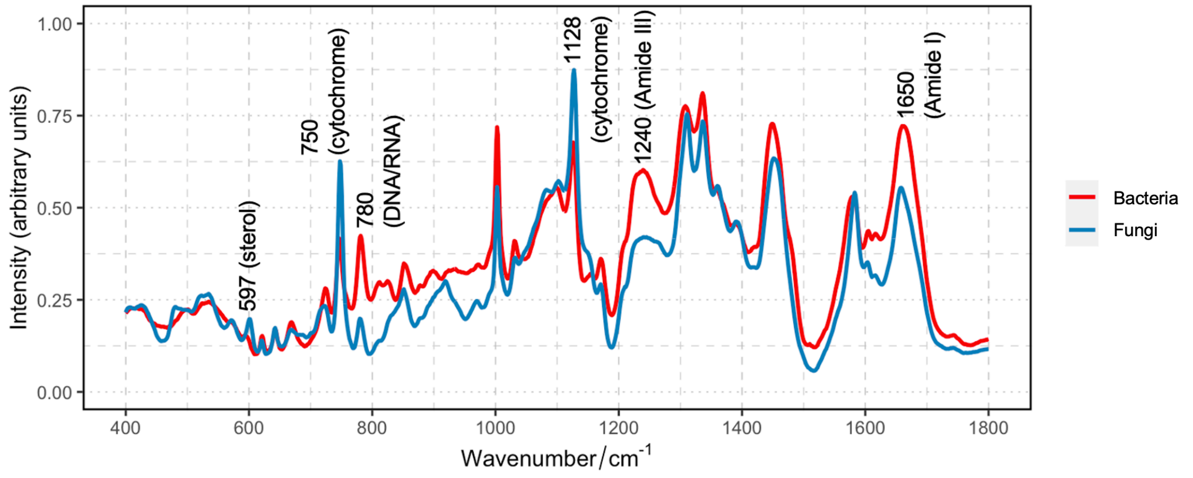

Supplementary Fig. S7 Raman spectra of bacteria and fungi. The average bacterial spectrum (red) was generated from n=74,400 single-cell Raman spectra (SCRS) from 248 bacterial strains, and the average fungal spectrum (blue) was generated from n=28,200 SCRS from 94 fungal strains. Fungal spectra show higher intensity traits related to cytochrome c at 750 cm^{-1} (pyrrole ring breathing) and 1128 cm^{-1} (C–N stretching) and ergosterol backbones at 597 cm^{-1}. In contrast, bacterial cells exhibit higher intensities in bands related to nucleic acids at 780 cm^{-1} (cytosine/uracil ring breathing) and proteins at 1240 cm^{-1} (Amide III) and 1650 cm^{-1} (Amide I).

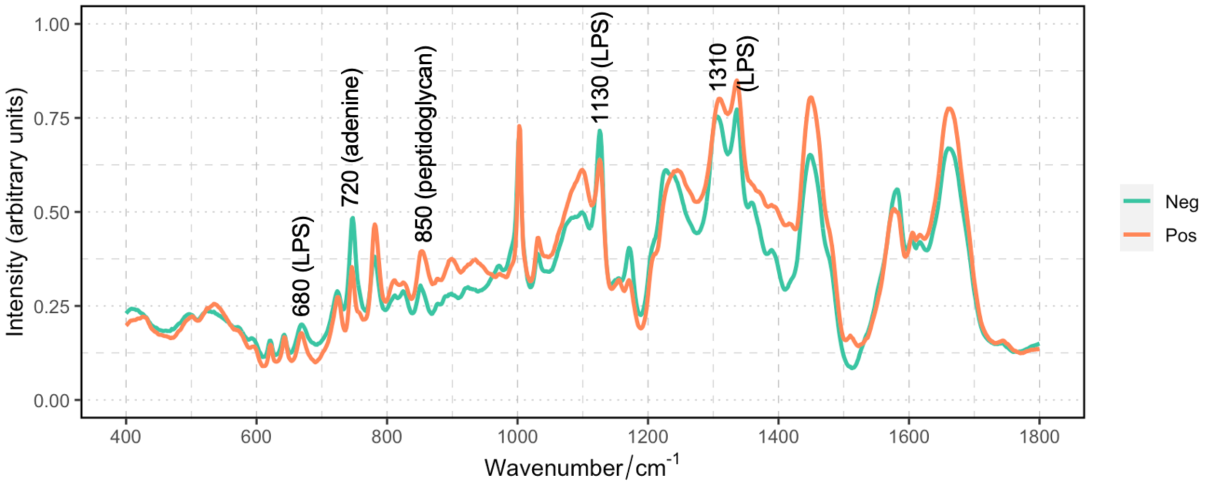

Supplementary Fig. S8 Raman spectra of Gram-negative and Gram-positive bacteria. The average Gram-positive spectrum (orange) was generated from n=24,300 SCRS from 81 strains, and the average Gram-negative spectrum (teal) was generated from n=50,100 SCRS from 167 strains. The distinction is mainly attributed to their unique cell wall components. Gram-positive bacteria exhibit bands due to their thicker peptidoglycan layer, notably around 850 cm^{-1}. Gram-negative bacteria show bands related to lipopolysaccharides (LPS) in their outer membrane, notably at 680, 1130, and 1310 cm^{-1}. Additional differences include bands at 720 cm^{-1} (adenine).

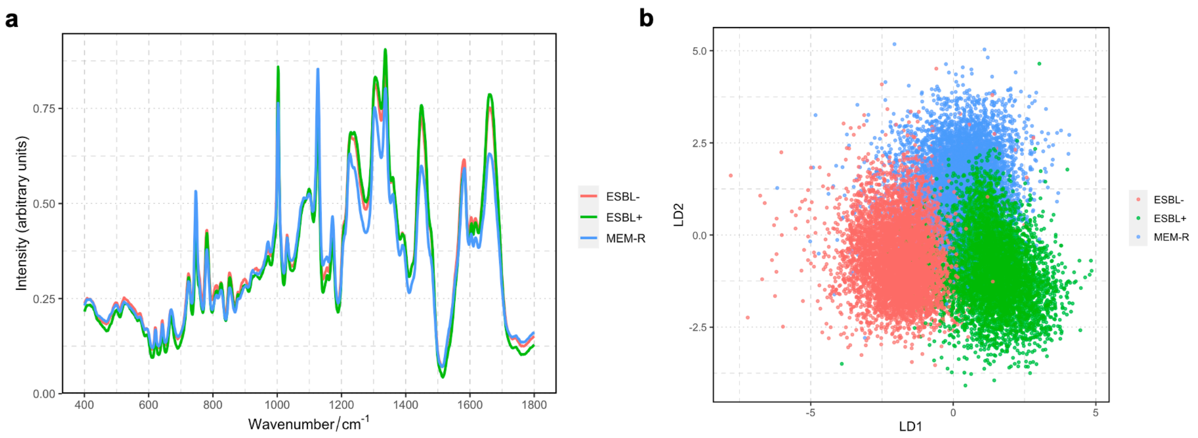

Supplementary Fig. S9 (**a**) Raman spectra of *E. coli* with different antibiotic resistance profiles: MEM-R (resistance to meropenem), ESBL+ (extended-spectrum beta-lactamase positive) and ESBL– (extended-spectrum beta-lactamase negative), each with four isolates. (**b**) linear discriminant analysis (LDA) showing a clear separation between single-cell spectra of cells with different resistance profiles. A total of n=1,200 spectra from 4 isolates were plotted for each of the three resistance profiles.

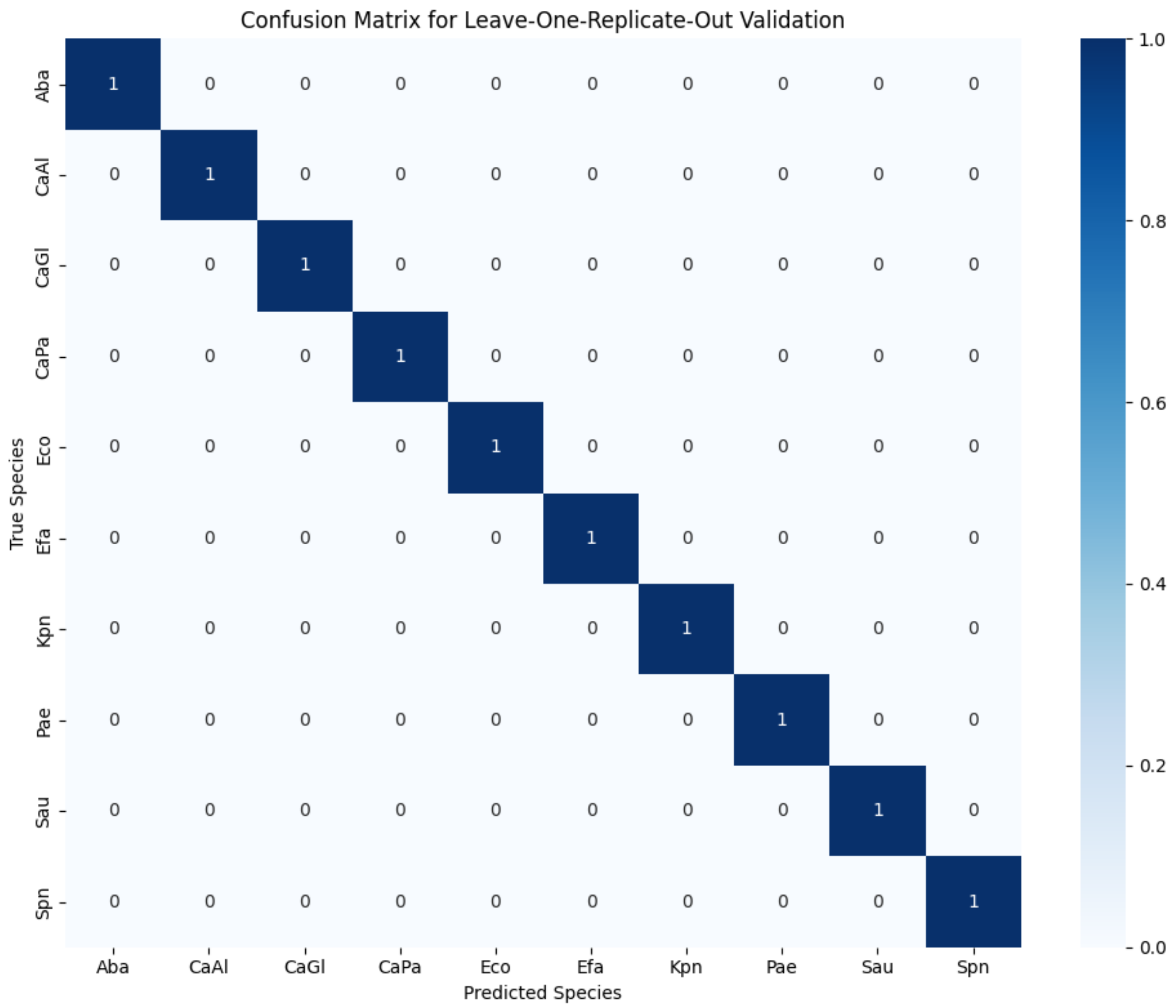

Supplementary Fig. S10 Leave-One-Replicate-Out Cross-Validation Results. Confusion matrix for Leave-One-Replicate-Out cross-validation using 9 independent biological replicates spanning representative Gram-negative bacteria (Aba, Eco, Pae), Gram-positive bacteria (Efa, Sau, Spn), and fungi (CaAl, CaGl, CaPa). Each replicate consisted of ~100 single-cell Raman spectra from an independent culture of the same clinical isolate used in the original database.

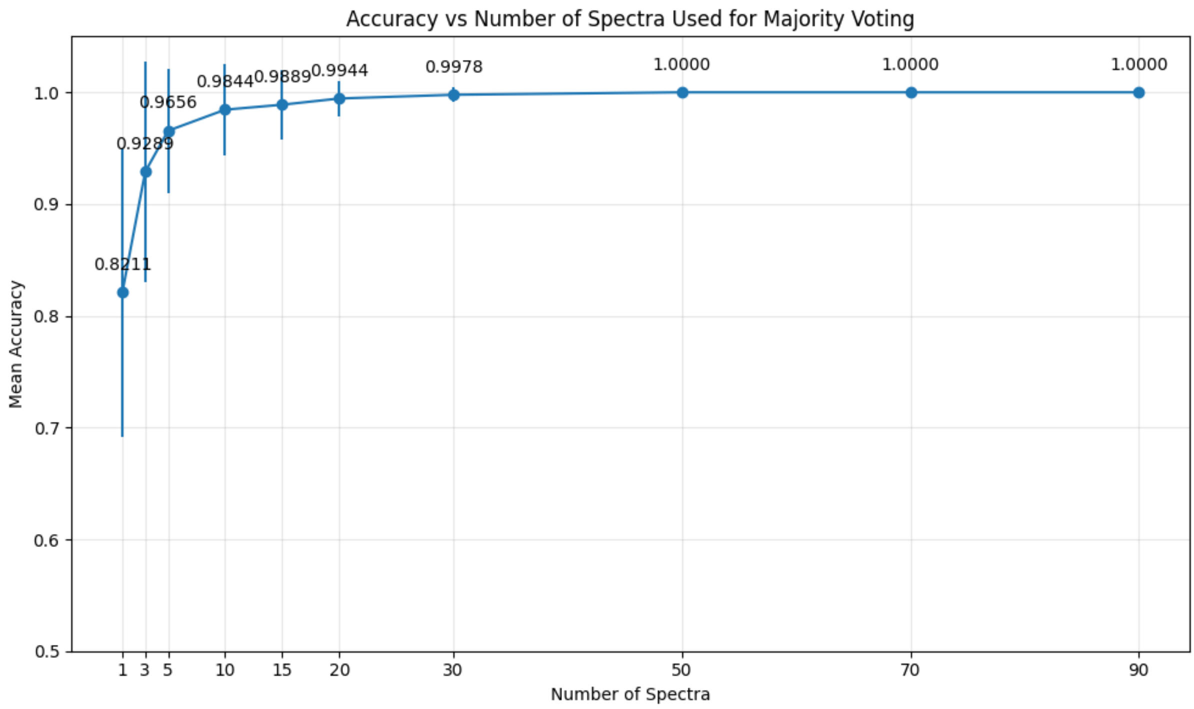

Supplementary Fig. S11 Bootstrap analysis demonstrating minimum spectra requirements for reliable pathogen identification. The analysis was performed using 9 independent biological replicates spanning representative Gram-negative bacteria (Aba, Eco, Pae), Gram-positive bacteria (Efa, Sau, Spn), and fungi (CaAl, CaGl, CaPa). For each replicate containing ~100 single-cell Raman spectra, random subsets of 1-90 spectra were sampled 100 times, and majority voting accuracy was calculated for each subset size. Error bars represent standard deviation across all replicates and bootstrap iterations.

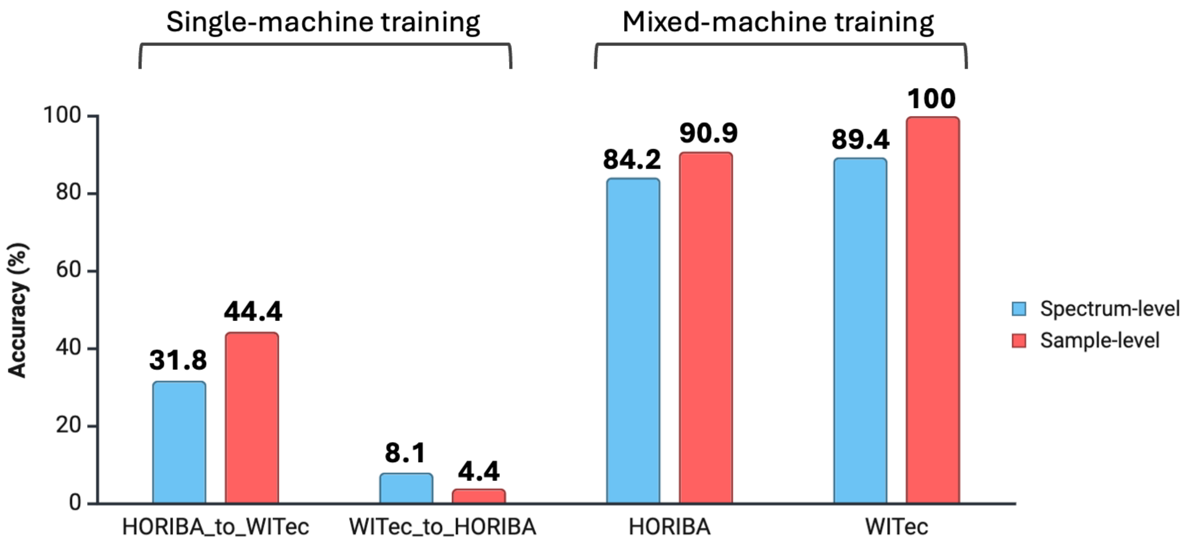

Supplementary Fig. S12 Cross-instrument performance comparison between single-machine and mixed-machine training approaches. The 'Mixed-machine training' accuracies were derived from a ten-times five-fold cross-validation procedure, and the bars represent the resulting mean accuracy. Spectrum-level accuracy represents the percentage of individual Raman spectra correctly classified, while sample-level accuracy represents the percentage of isolates correctly identified based on majority voting from multiple spectra per isolate. Single-machine training shows poor transferability, with HORIBA-to-WITec achieving 31.8% spectrum-level and 44.4% sample-level accuracy, while WITec-to-HORIBA shows even lower performance (8.1% and 4.4%, respectively). In contrast, mixed-machine training demonstrates robust cross-instrument performance, achieving 84.2% spectrum-level accuracy for HORIBA and 89.4% for WITec instruments, with corresponding sample-level accuracies of 90.9% and 100%, respectively.

Patient ID	Sample Type
H1	Blood
H2	Blood
H3	Blood
H4	Blood
H5	Blood
H11	Blood
H12	Blood
H16	Blood
H17	Blood
H18	Blood
H19	Blood
H20	Blood
S003	Blood
S004	Blood

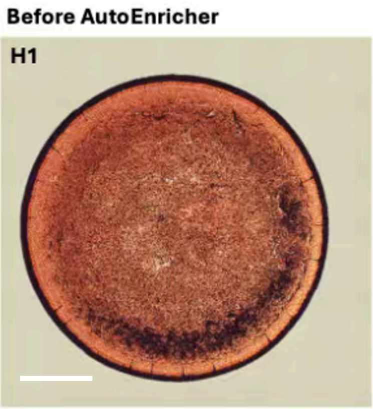

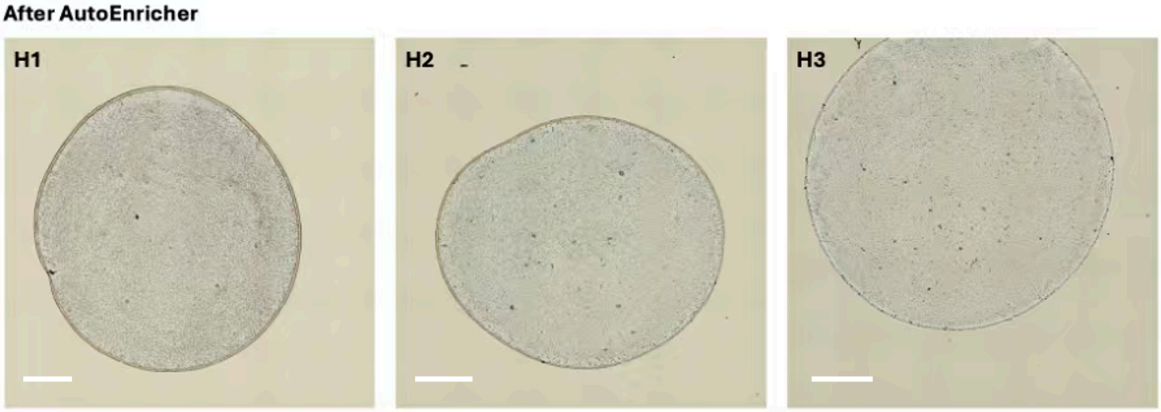

Supplementary Fig. S13. AutoEnricher processing of blood culture samples demonstrates effective matrix cleanup and pathogen enrichment. The table shows the 15 positive blood culture samples collected from patients at two hospitals. Representative microscopic images show blood culture samples before and after AutoEnricher processing. **Before AutoEnricher**: Blood culture sample (H1) shows dense, complex matrix with high background of blood cells that would interfere with single-cell Raman spectroscopy. **After AutoEnricher**: Processed samples (H1-H3) demonstrate effective removal of blood cell background and matrix components, resulting in clean samples suitable for single-cell analysis. These images are representative of all 15 samples, and the cleaning process was technically validated three times with similar results. Scale bar: 500 μm.

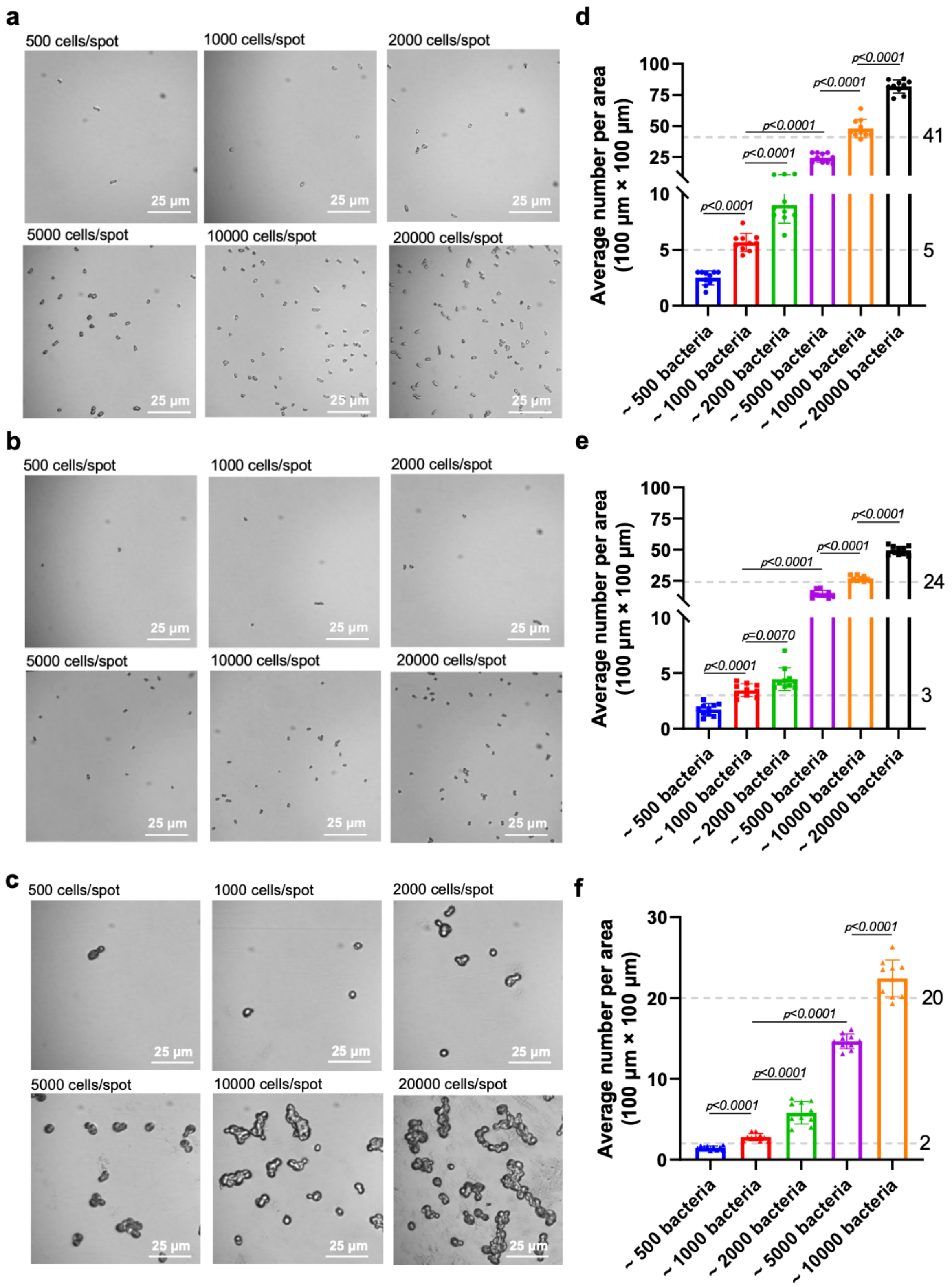

Supplementary Fig. S14 Pathogen density thresholds for visual detection of positive/negative infection. (a-c) Representative microscopic images from three experiments showing pathogen density gradients for **(a)** *E. coli* (rod-shaped), **(b)** *E. faecalis* (cocci-shaped) at concentrations ranging from 500 to 20000 cells and **(c)** *Candida auris* (fungi) at

concentrations ranging from 500 to 20000 cells per 2 µL spot. Scale bars: 25 µm. **(d-f)** Quantitative analysis of bacterial density per defined area (100 µm × 100 µm) for **(d)** *E. coli*, **(e)** *E. faecalis* and **(f)** *C. auris*. Horizontal dashed lines indicate threshold values (mean-SD) for distinguishing >1000 pathogens (5 bacteria/area or 500/mm^2 for *E. coli*, 3 bacteria/area or 300/mm^2 for *E. faecalis*, 2 fungi/area or 200/mm^2 for *C. auris*). Data represent mean ± SD from analysis of 10 random areas from two biological replicates. Statistical significance determined by one-tailed unpaired t-test. Source data for (d), (e) and (f) are provided as a Source Data file.

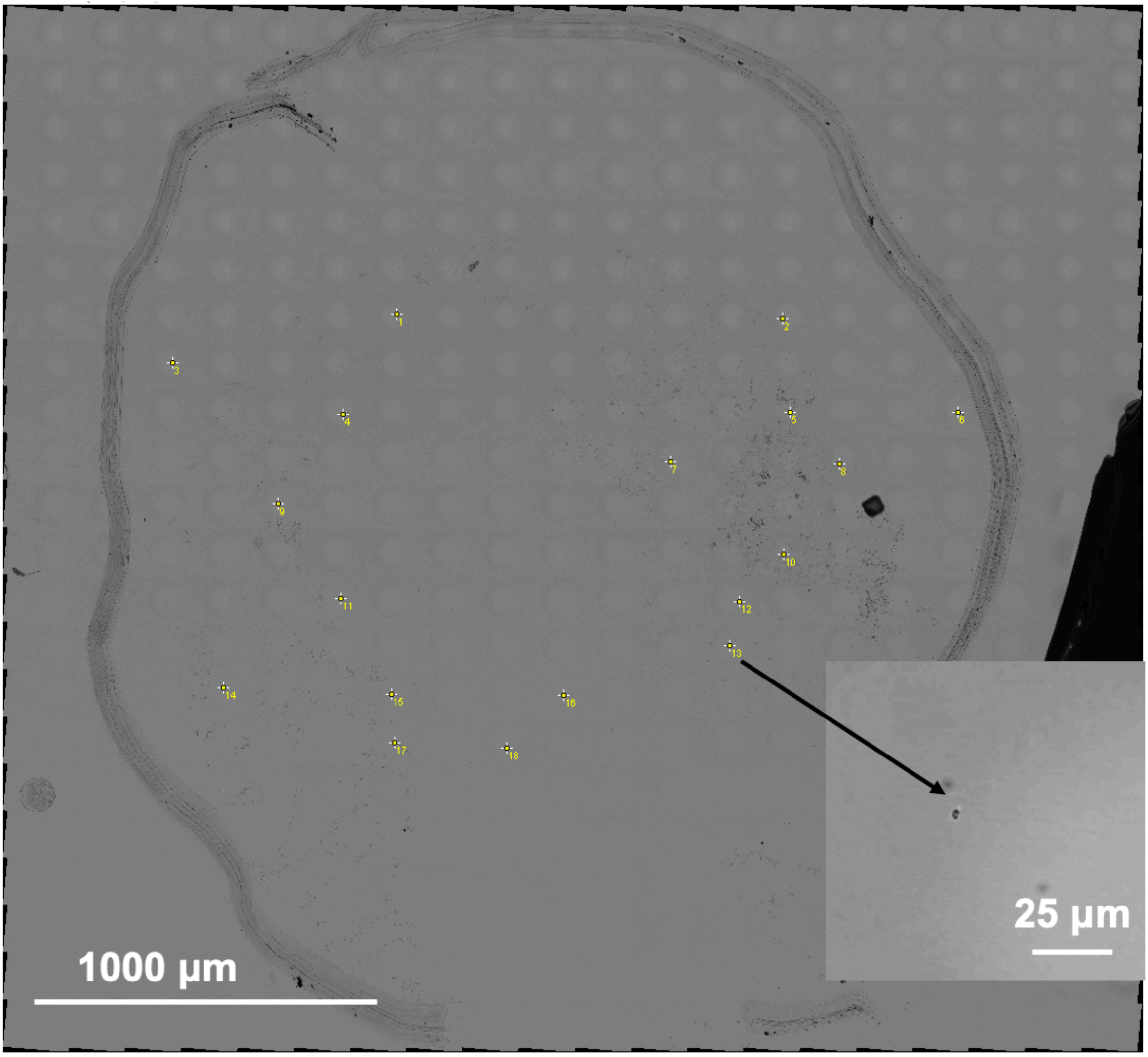

Supplementary Fig. S15 Extremely low-concentration detection example showing tiled imaging approach for detecting individual bacteria in 2-µl post-enrichment sample containing ~20 *E. coli* cells. Yellow markers indicate identified bacterial cells (n = 18) across 440 tiled images. Scale bars: 1000 µm (overview), 25 µm (detail).

a

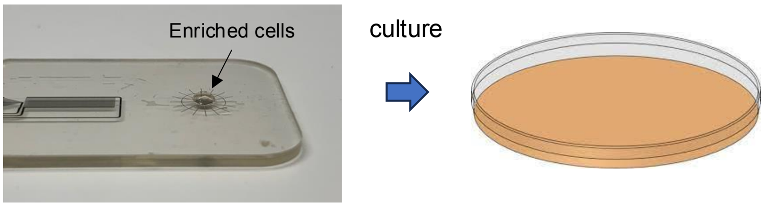

b

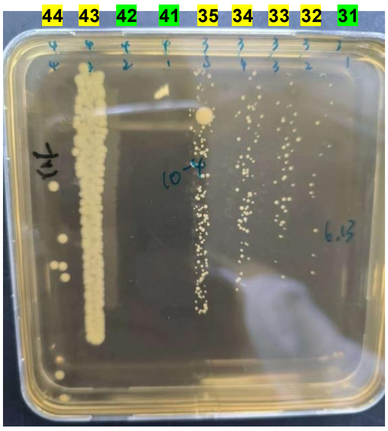

ID	Sample	[a]Time (min)	[b]Cell number
[c]31	N-Control-1	5	0
32	H11	1	3.9×10^4
33	H11	3	1.2×10^5
34	H11	5	2.2×10^5
35	H11	10	3.9×10^5
[c]41	N-Control-2	5	0
[d]42	H12-control	5	0
43	H12	0.5	2.1×10^4
44	H12	10	3.3×10^5

[a]: enriched duration
[b]: total number of cells in the droplets
[c]: negative control. Only DI water runs through devices
[d]: cells trapped without release.

c

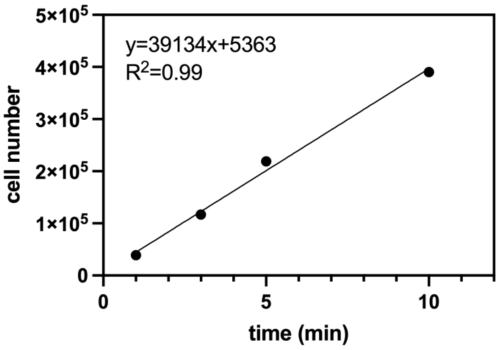

Supplementary Fig. S16 (**a**) Plate culturing of isolated pathogens from the AutoEnricher. 3 μL of the droplet at the opening of the DEP chip was collected and diluted for agar plate culturing to determine cell number. (**b**) two clinical samples (H11 and H12) were enriched for various durations to evaluate the number of captured cells. Left: images of agar culture; Right: details of sample ID and cell counts. For each device, a negative control was conducted first, using DI water as the sample solution. Typically, after capturing pathogens from the sample, the system was switched to DI water to remove the residue matrix. To ensure no cells were lost during this process, an additional control was conducted with Sample H12, where the enriched pathogens were not released for droplet collection. (**c**) The enriched cell number increased linearly with capture duration (Sample 11), demonstrated the high capture efficiency of our approach. Sample 11 was identified as *Staphylococcus capitis*, and Sample H12 as *E. coli*, using MALDI-TOF. Source data for (c) are provided as a Source Data file.

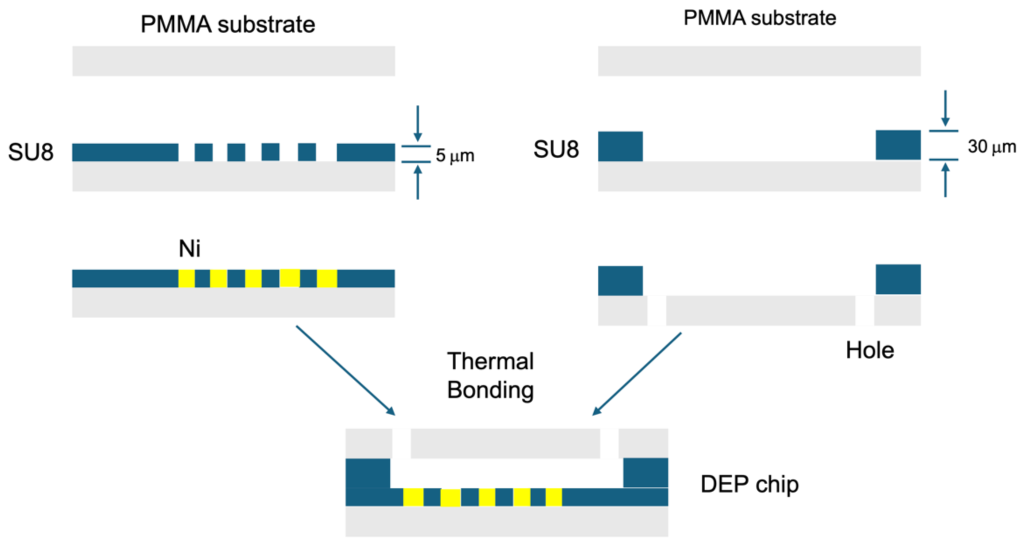

Supplementary Fig. S17 Schematic illustration of the DEP chip fabrication. The DEP chip was fabricated using two 500 µm PMMA substrates. The left side illustrates the fabrication steps for creating nickel electrodes on one PMMA substrate, while the right side depicts the steps for forming the microfluidic channel on the other PMMA substrate. The two PMMA substrates were thermally bonded at 100°C to form an enclosed DEP chip.
