# Supplementary material for: Rapid culture-free diagnosis of clinical pathogens via integrated microfluidic-Raman micro-spectroscopy
Source: Nat Commun. 2025 Dec 16;17:283. doi: 10.1038/s41467-025-66996-y (PMC12783191; doi:10.1038/s41467-025-66996-y)
Supplement: Supplementary file 3 — Description of Additional Supplementary Files [file 41467_2025_66996_MOESM3_ESM.pdf]

Description of Additional Supplementary Files:

Supplementary Data 1. List of species abbreviations for 36 bacterial and fungal species.

Supplementary Data 2. Comprehensive list of all 342 clinical isolates used in the study.

Supplementary Data 3. Antibiotic resistance profiles for 12 E. coli isolates.

Supplementary Data 4. Details of the 40 patient samples used for the fine-tuning dataset.

Supplementary Data 5. Clinical validation data for the 305-patient cohort, including details of the 120 samples used for Raman identification.

Supplementary Movie 1. Real-time trapping of RFP E. coli cells on the IDT array. Concentration of cells: ~ 50 CFU/mL; flow rate: 200 mL/min; voltage: 20V. AC power was turned on at the beginning of the recording and turned off at the end of the Movie.

Supplementary Movie 2. Real-time trapping of RFP E. coli cells on the IDT array. Concentration of cells: ~ 2000 CFU/mL; Flow rate: 200 mL/min; Voltage: 40V. AC power was applied at 5s and released at 185s.

Supplementary Movie 3. Real-time trapping of Klebsiella oxytoca cells on the IDT array. Concentration of cells: ~ 5000 CFU/mL; flow rate: 200 mL/min; voltage: 40V. AC power was turned on at the beginning of the recording and was turned off at the end of the Movie. AC power was applied at 5s and released at 125s.

Supplementary Movie 4. Real-time trapping of Klebsiella aerogenes cells on the IDT array. Concentration of cells: ~ 5000 CFU/mL; flow rate: 200 mL/min; voltage: 40V. AC power was applied at 5s and released at 125s.

Supplementary Movie 5. Real-time trapping of Klebsiella pneumoiniae cells on the IDT array. Concentration of cells: ~ 5000 CFU/mL; flow rate: 200 mL/min; voltage: 40V. AC power was applied at 5s and released at 125s.

Supplementary Movie 6. Real-time trapping of Enterococcus faecalis cells on the IDT array. Concentration of cells: ~ 5000 CFU/mL; flow rate: 200 mL/min; voltage: 40V. AC power was applied at 5s and released at 125s.

Supplementary Movie 7. Real-time trapping of Enterococcus faecium cells on the IDT array. Concentration of cells: ~ 5000 CFU/mL; flow rate: 200 mL/min; voltage: 40V. AC power was applied at 5s and released at 125s.

Supplementary Movie 8. Real-time trapping of Staphylococcus capitis cells on the IDT array. Concentration of cells: ~ 5000 CFU/mL; flow rate: 200 mL/min; voltage: 40V. AC power was applied at 5s and released at 125s.

Supplementary Movie 9. Real-time trapping of Candida albicans cells on the IDT array. Concentration of cells: ~ 5000 CFU/mL; flow rate: 200 mL/min; voltage: 40V. AC power was applied at 5s and released at 125s.

Supplementary Movie 10. Real-time trapping of Candida glabrata cells on the IDT array. Concentration of cells: ~ 5000 CFU/mL; flow rate: 200 mL/min; voltage: 40V. AC power was applied at 5s and released at 125s.

Supplementary Movie 11. Real-time trapping of Candida parapsilosis cells on the IDT array. Concentration of cells: ~ 5000 CFU/mL; flow rate: 200 mL/min; voltage: 40V. AC power was applied at 5s and released at 125s.
