# Supplementary material for: Rapid culture-free diagnosis of clinical pathogens via integrated microfluidic-Raman micro-spectroscopy
Source: Nat Commun. 2025 Dec 16;17:283. doi: 10.1038/s41467-025-66996-y (PMC12783191; doi:10.1038/s41467-025-66996-y)
Supplement: Supplementary file 20 — Reporting Summary [file 41467_2025_66996_MOESM20_ESM.pdf]

nature portfolio

Corresponding author(s): *Double-anonymous peer review submissions: write DAPR and your manuscript number here instead of author names.*

Last updated by author(s): *YYYY-MM-DD*

Reporting Summary

Nature Portfolio wishes to improve the reproducibility of the work that we publish. This form provides structure for consistency and transparency in reporting. For further information on Nature Portfolio policies, see our Editorial Policies and the Editorial Policy Checklist.

Statistics

For all statistical analyses, confirm that the following items are present in the figure legend, table legend, main text, or Methods section.

n/a	Confirmed	
☐	☒	The exact sample size (n) for each experimental group/condition, given as a discrete number and unit of measurement
☐	☒	A statement on whether measurements were taken from distinct samples or whether the same sample was measured repeatedly
☐	☒	The statistical test(s) used AND whether they are one- or two-sided<br>*Only common tests should be described solely by name; describe more complex techniques in the Methods section.*
☒	☐	A description of all covariates tested
☒	☐	A description of any assumptions or corrections, such as tests of normality and adjustment for multiple comparisons
☐	☒	A full description of the statistical parameters including central tendency (e.g. means) or other basic estimates (e.g. regression coefficient) AND variation (e.g. standard deviation) or associated estimates of uncertainty (e.g. confidence intervals)
☒	☐	For null hypothesis testing, the test statistic (e.g. F, t, r) with confidence intervals, effect sizes, degrees of freedom and P value noted<br>*Give P values as exact values whenever suitable.*
☒	☐	For Bayesian analysis, information on the choice of priors and Markov chain Monte Carlo settings
☒	☐	For hierarchical and complex designs, identification of the appropriate level for tests and full reporting of outcomes
☒	☐	Estimates of effect sizes (e.g. Cohen's d, Pearson's r), indicating how they were calculated

Our web collection on statistics for biologists contains articles on many of the points above.

Software and code

Policy information about availability of computer code

Data collection	AutoEnricher control: Custom Python software on Raspberry Pi 4b; Raman spectroscopy: HORIBA LabSpec 6 and WITec Control software; Microscopy: Zeiss ZEN software for fluorescence imaging
Data analysis	Preprocessing: R 4.2.2; Deep learning: Python with TensorFlow/Keras for 1D ResNet model; Statistical analysis: R 4.2.2, GraphPad Prism 8.0.2; Image analysis: ImageJ 1.48; Data visualization: Python matplotlib, R ggplot2; All custom code and datasets available at: https://osf.io/784pz/ (DOI 10.17605/OSF.IO/784PZ)

For manuscripts utilizing custom algorithms or software that are central to the research but not yet described in published literature, software must be made available to editors and reviewers. We strongly encourage code deposition in a community repository (e.g. GitHub). See the Nature Portfolio guidelines for submitting code & software for further information.

Data

Policy information about availability of data

All manuscripts must include a data availability statement. This statement should provide the following information, where applicable:

- Accession codes, unique identifiers, or web links for publicly available datasets
- A description of any restrictions on data availability
- For clinical datasets or third party data, please ensure that the statement adheres to our policy

All datasets are available at https://osf.io/784pz/; DOI 10.17605/OSF.IO/784PZ

Research involving human participants, their data, or biological material

Policy information about studies with human participants or human data. See also policy information about sex, gender (identity/presentation), and sexual orientation and race, ethnicity and racism.

Reporting on sex and gender	Not applicable. Sex and gender data not collected. Study focused on pathogen identification from clinical samples rather than patient demographics.
Reporting on race, ethnicity, or other socially relevant groupings	Not applicable. Study analysed microbial pathogens from clinical samples.
Population characteristics	Study involved surplus clinical samples from 305 patients across three hospitals in China (Peking Union Medical College Hospital, Huashan Hospital Shanghai, Sir Run Run Shaw Hospital Zhejiang). Patient demographics not recorded - only sample types and infection status relevant to diagnostic validation.
Recruitment	Consecutive surplus clinical samples collected from routine hospital microbiology workflows. No direct patient recruitment.
Ethics oversight	Peking Union Medical College Hospital Ethics Committee (No. S-K676); Huashan Hospital Ethics Committee (No. 2020-907); Sir Run Run Shaw Hospital Ethics Committee (No. 20200316-33)

Note that full information on the approval of the study protocol must also be provided in the manuscript.

Field-specific reporting

Please select the one below that is the best fit for your research. If you are not sure, read the appropriate sections before making your selection.

[x] Life sciences [ ] Behavioural & social sciences [ ] Ecological, evolutionary & environmental sciences

For a reference copy of the document with all sections, see nature.com/documents/nr-reporting-summary-flat.pdf

Life sciences study design

All studies must disclose on these points even when the disclosure is negative.

Sample size	Clinical validation: 305 patients determined by consecutive sample collection during study period. Raman identification subset: 120 samples calculated using power analysis - minimum 61 samples required (80% target accuracy, 95% confidence, 10% margin of error), expanded to 120 with safety buffer. Database construction: 342 clinical isolates across 36 species to ensure representative coverage of major hospital pathogens.
Data exclusions	Blood culture samples (n=15) excluded from 305-sample culture-free validation due to regulatory requirement for collection in culture media, inconsistent with culture-free workflow. Quality control exclusions: abnormal/burnt high-intensity Raman spectra and cosmic ray artifacts removed during preprocessing. No other data exclusions applied.
Replication	Capture efficiency experiments: minimum n=3 independent replicates per condition. Raman database: 300 spectra per isolate from 3 independent culture batches. Cross-validation: 5-fold stratified validation with 100 Monte Carlo trials. Leave-One-Replicate-Out validation: 9 species tested with independent biological replicates. Bootstrap analysis: 100 iterations per spectrum count. All key findings successfully replicated across experiments.
Randomization	Not applicable for clinical validation - surplus samples used. Database construction: random spectrum selection for validation sets. Cross-validation: stratified random splitting ensuring isolate separation between training/validation sets.
Blinding	Raman identification analysis fully blinded - 120 clinical samples processed and analyzed without knowledge of culture/MALDI-TOF results until comparison phase.

Reporting for specific materials, systems and methods

We require information from authors about some types of materials, experimental systems and methods used in many studies. Here, indicate whether each material, system or method listed is relevant to your study. If you are not sure if a list item applies to your research, read the appropriate section before selecting a response.

Materials & experimental systems

n/a	Involved in the study
☒	☐ Antibodies
☒	☐ Eukaryotic cell lines
☒	☐ Palaeontology and archaeology
☒	☐ Animals and other organisms
☐	☒ Clinical data
☒	☐ Dual use research of concern
☒	☐ Plants

Methods

n/a	Involved in the study
☒	☐ ChIP-seq
☒	☐ Flow cytometry
☒	☐ MRI-based neuroimaging

Clinical data

Policy information about clinical studies
All manuscripts should comply with the ICMJE guidelines for publication of clinical research and a completed CONSORT checklist must be included with all submissions.

Clinical trial registration	Not applicable. Study involved diagnostic validation using surplus clinical samples, not a clinical trial requiring registration.
Study protocol	Full protocols available in Methods section and Supplementary Information. Ethics approvals obtained from all participating institutions (PUMCH S-K676, Huashan 2020-907, Sir Run Run Shaw 20200316-33) for use of anonymized surplus clinical samples.
Data collection	Settings: Three hospitals in China - Peking Union Medical College Hospital (Beijing), Huashan Hospital (Shanghai), Sir Run Run Shaw Hospital (Zhejiang). Recruitment period: Consecutive sample collection during study period.
Outcomes	Primary: Diagnostic accuracy of AutoEnricher for infection status determination (sensitivity/specificity vs. culture reference). Secondary: Species identification accuracy using Raman spectroscopy vs. MALDI-TOF MS reference standard. Assessed by blinded comparison of AutoEnricher/Raman results with hospital culture and MALDI-TOF identification results.

Plants

Seed stocks	*Report on the source of all seed stocks or other plant material used. If applicable, state the seed stock centre and catalogue number. If plant specimens were collected from the field, describe the collection location, date and sampling procedures.*
Novel plant genotypes	*Describe the methods by which all novel plant genotypes were produced. This includes those generated by transgenic approaches, gene editing, chemical/radiation-based mutagenesis and hybridization. For transgenic lines, describe the transformation method, the number of independent lines analyzed and the generation upon which experiments were performed. For gene-edited lines, describe the editor used, the endogenous sequence targeted for editing, the targeting guide RNA sequence (if applicable) and how the editor was applied.*
Authentication	*Describe any authentication procedures for each seed stock used or novel genotype generated. Describe any experiments used to assess the effect of a mutation and, where applicable, how potential secondary effects (e.g. second site T-DNA insertions, mosiacism, off-target gene editing) were examined.*
